# Efficacy and Safety of Polyherbal Formulation in Low Sperm Count Participants: A Randomized, Multicenter, Double-Blinded, Placebo-Controlled, Interventional Study

**DOI:** 10.7759/cureus.98844

**Published:** 2025-12-09

**Authors:** Pramod Kolsure, Sanman Kolhe, Aditya Dev, Somesh S Shintre, Shrikant G Dhavale, Vinod Kuber, Sriram Padmanabhan

**Affiliations:** 1 Formulation and Development, Formulation Development Team, SAVA Healthcare Limited, Pune, IND; 2 Research and Development, Innovation and Drug Discovery Team, SAVA Healthcare Limited, Pune, IND; 3 Research and Development, Formulation Development Team, SAVA Healthcare Limited, Pune, IND

**Keywords:** ashwagandha, male infertility, oligospermia, sexual health, sperm count

## Abstract

Introduction: Male infertility, particularly oligospermia, is characterized by reduced sperm count. It remains a significant clinical concern due to its multifactorial origin and limited therapeutic options. The present study investigates the efficacy and safety of “SHL 1066,” a standardized polyherbal formulation, in enhancing semen quality and improving sexual health outcomes in men with low sperm counts.

Materials and methods: In a 90-day, multicenter, randomized, double-blinded, placebo-controlled trial, 60 male participants were randomly assigned to receive either “SHL 1066” or a placebo using a computer-generated randomization protocol. Assessments of sperm count, motility, serum total testosterone, and sexual health parameters (erectile function, intercourse satisfaction, and overall satisfaction) were conducted at baseline and study completion.

Results: After 90 days, “SHL 1066” significantly increased sperm count by 72.5% and motility by 14.4%, compared to 8.6% and 6.3%, respectively, in the placebo group. Serum total testosterone levels rose by 23.3%. Sexual health outcomes improved markedly, with erectile function enhanced by 45.5%, intercourse satisfaction by 52.8%, and overall satisfaction by 54%, substantially surpassing placebo outcomes.

Conclusion: “SHL 1066” demonstrated safety, tolerability, and significant efficacy in improving sperm count, motility, testosterone levels, and sexual health compared to placebo. These results position “SHL 1066” as a promising therapeutic option for managing male infertility by enhancing semen quality and sexual function.

## Introduction

The World Health Organization (WHO) defines male infertility as a man's inability to cause pregnancy in a fertile female after at least one year of regular, unprotected intercourse. In approximately 20% of infertility cases, the male is solely responsible, and in another 30-40%, he is a contributing factor [1]. Male-related infertility is commonly associated with conditions such as oligospermia (reduced sperm count), difficulties in semen release, or abnormalities in sperm morphology and motility [2].

Oligospermia, or oligozoospermia, represents one of the most common causes of male infertility and is defined by the WHO as a sperm concentration of fewer than 15 million sperm per milliliter of semen [3,4]. Azoospermia refers to the complete lack of sperm in the seminal fluid. This condition affects approximately 1% of all men and 10-15% of those who are infertile [5]. Teratozoospermia involves abnormal sperm morphology affecting various parts of the sperm. Oligoasthenoteratozoospermia, a frequent infertility condition, involves abnormalities in sperm number, movement, and morphology [6].

Male infertility, particularly oligospermia, often stems from dysfunctions within the hypothalamic-pituitary-gonadal (HPG) axis, which governs gonadal and sexual function via complex hormonal feedback mechanisms. Testicular activity is regulated by the hypothalamus via pulsatile gonadotropin-releasing hormone (GnRH) secretion, which stimulates testosterone synthesis in Leydig cells. Testosterone is essential for sustaining spermatogenesis in the seminiferous tubules. Disruptions in the HPG axis, due to elevated prolactin, stress, systemic illness, or exogenous hormones, can impair GnRH secretion, reduce testosterone synthesis, and negatively impact sperm production. Spermatogenesis, a tightly regulated process occurring over approximately 74 days, involves the transformation of germ cells through mitotic and meiotic divisions and spermiogenesis. Impairments in hormonal balance, Leydig cell function, genetic factors, or obstruction of sperm transport pathways can compromise sperm quality and quantity, contributing to male infertility [7].

Male infertility can stem from pretesticular (hormonal issues like Kallman syndrome, pituitary disorders, thyroid imbalances), testicular (e.g., Klinefelter syndrome, cryptorchidism, varicocele), or post-testicular causes (e.g., vas deferens absence, infections, ejaculatory dysfunction) [8].

Many treatment options, including surgery (e.g., varicocelectomy), hormonal and drug therapy [9], intrauterine insemination, and in vitro fertilization, intracytoplasmic sperm injection, and assisted reproductive technology methods, are available [10]. However, these may have limited effectiveness and potential side effects [11,12]. To date, idiopathic oligospermia remains challenging to treat, with no definitive cure. Traditional medicine systems like Ayurveda have shown promising effects using herbs such as *Asparagus adscendens, Withania somnifera, Tribulus terrestris, and Asparagus racemosus*. This indicates that an ayurvedic medication alternative can be a better treatment option for treating oligospermia [13-23]. 

Taking leads from these effective alternative methods, such as Ayurveda, Sava Lifesciences Inc. has developed “SHL 1066,” an Ayurvedic Proprietary Medicine containing *Asparagus adscendens* [13,14], *Withania somnifera *[15,16], *Tribulus terrestris* [17-19], *Asparagus racemosus* [20,21], and *Emblica officinalis* [22,23] to effectively manage oligospermia. Based on existing literature, it was hypothesized that SHL 1066 may improve sperm quality and overall reproductive function in men with oligospermia. For this study, sperm count was defined as the primary endpoint, while sperm motility, sperm morphology, serum testosterone levels, and International Index of Erectile Function (IIEF) scores were designated as secondary endpoints. Accordingly, a randomized, multicenter, double-blinded, placebo-controlled clinical study was conducted to evaluate the efficacy and safety of SHL 1066 in patients with low sperm count.

## Materials and methods

Study design

This was a randomized, multicenter, double-blinded, placebo-controlled, interventional, prospective, clinical study for the evaluation of the efficacy and safety of “SHL 1066” in patients with low sperm count. The study groups received either of the following treatments: The test group received a “SHL 1066,” and the placebo group received a placebo tablet. The duration of the treatment period was 90 days. The study was initiated only after written approval was obtained from the Independent Ethics Committee, Dhanashree Hospital, Navi Sangvi, Pune, and the Ethics Committee MES Ayurved Mahavidyalaya, and subsequent registration of the study on the Clinical Trial Registry-India (CTRI) website. The study was conducted as per approved protocol and as per Good Clinical Practices guidelines-AYUSH. The clinical trial was registered with the CTRI under the registration number CTRI/2018/10/016031 (Registered on: 15/10/2018). Participants were enrolled in the study. The study was conducted on three sites: Aniruddha Clinic, Shirpur, Dhule; MES Ayurved Mahavidyalaya, Ratnagiri; and Jyoti Multispecialty Clinic, Kalewadi, Pune. The study was initiated only after written approval was obtained from the Independent/Institutional Ethics Committee and subsequent registration of the study on the CTRI website. The randomization sequence was generated by an independent statistician. Site investigators enrolled eligible participants. Allocation was implemented using sequentially numbered, identical, sealed containers. Study staff responsible for outcome assessments remained blinded to group assignment throughout the study.

Investigational product details

“SHL 1066”-Polyherbal Formulation is the product code used in the study, i.e., “SHL 1066,” which is GAMETE tablet (Food and Drug Administration-approved product). Each tablet included Shwet Musali (*Asparagus adscendens*) extract, Ashwagandha (*Withania somnifera*) extract, Gokshur (*Tribulus terrestris*) extract, Shatavari (*Asparagus racemosus*) extract, and Amalaki (*Emblica officinalis*) extract. The placebo tablet was made up of inert ingredients. The placebo was manufactured to match the active tablet in appearance, shape, size, and color to ensure organoleptic indistinguishability. Both SHL 1066 and placebo tablets were packaged in identical containers to maintain blinding.

Inclusion criteria

Married men aged 21 to 45 years of age (both inclusive) and those with a sperm count of one million to less than 15 million/ml with normal sperm morphology were included in the study. The subjects are capable of giving written informed consent, which includes compliance with the protocol. Subjects who agreed not to use any other treatment for erectile dysfunction (ED), those who were willing to give a specimen of semen before and at the end of the clinical trial, and subjects who were willing to observe physical abstinence as per the protocol requirement (i.e., one week before semen specimen) were included in the study.

Exclusion criteria

Exclusion criteria included known causes of male infertility (e.g., azoospermia, aspermia, necrospermia, varicocele, hydrocele, undescended testis, inguinal hernia), accessory gland infections, IIEF-EF >26, prior nonresponse to phosphodiesterase type 5 (PDE5) inhibitors, recent fertility treatments, untreated endocrine disorders, relevant surgical history, penile abnormalities, or medications affecting sexual function. Subjects with significant comorbidities, substance abuse, heavy tobacco use (>10/day), abnormal labs/ECG, recent or planned surgery, recent clinical trial participation, recent medication use (including traditional), allergies to study components, or inability to abstain from sexual activity prior to semen collection were also excluded.

Sample size

Sample size calculation was derived based on the primary end point, i.e., change in the total number of sperm cells per milliliter of seminal fluid from baseline to three months. Based on the assumptions that the mean difference in sperm cell count in SHL 1066 and placebo would be 11.96 with a desired precision of 5%, a total of 60 completed cases (30 in each group) were needed to assess the study objective at 80% power and 5% level of significance. The software used for the calculation of sample size was SPSS Version 10 (Released 1999; SPSS Inc., Chicago, Illinois, United States).

Methodology

**Figure 1:**
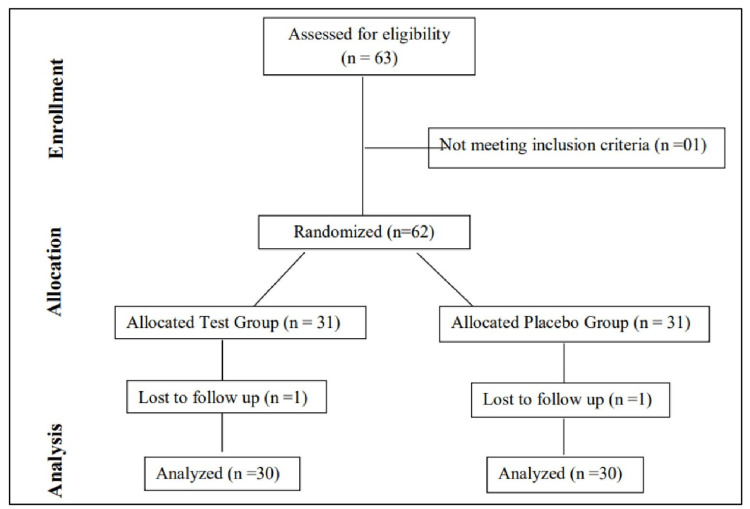
CONSORT diagram for the study CONSORT: Consolidated Standards of Reporting Trials

This was a randomized, multicenter, double-blinded, placebo-controlled, interventional, prospective, clinical study for the evaluation of the efficacy and safety of “SHL 1066” in patients with low sperm count. The tablet formulation contained standardized herbal extracts known for their potential benefits in male reproductive health. Subject disposition is shown in Figure 1. 

Following Ethics Committee approval and CTRI registration, married male subjects aged 21-45 years attending the outpatient department were screened. After obtaining written informed consent, demographic data, medical/surgical history, current medications, and vital signs were recorded. Subjects underwent clinical examination. Semen analysis was conducted after at least one week of sexual abstinence.

Eligible subjects returned fasting the next day for laboratory investigations, including testosterone levels, complete blood count (CBC), erythrocyte sedimentation rate (ESR), Hb%, fasting blood sugar level (BSL-F), liver function tests (LFTs), lipid and renal profiles, HIV I and II, chest X-ray, urine analysis, ECG, and testicular USG if required. Use of antioxidant agents, vitamins, hormonal, or herbal therapies was restricted throughout the study. A 14-day screening window was maintained to accommodate testing delays.

At baseline (Day 0), the subjects meeting all the inclusion criteria were randomly allocated (as per a computer-generated randomization list) to either one of the two treatment arms in a 1:1 ratio. Subjects were randomized to either the “SHL 1066” group or the placebo group with a dose of two tablets twice daily with water after a meal for 90 days.

Subjects were instructed to maintain their regular diet and exercise, avoid restricted medications, and report any adverse events. Compliance was monitored at each monthly follow-up; those missing >3 consecutive days or >9 total days of dosing were considered dropouts.

Monthly follow-ups were conducted for three months, with clinical examination, vital signs, and assessment of erectile function (IIEF). At the final visit (Day 90), semen analysis (after abstinence), hormone profiling, and repeat lab tests were performed. Tolerability was assessed by both the investigator and the subject, and adverse events were closely monitored throughout. Post-study, all subjects were advised to discontinue study medication and consult the investigator for further care.

Statistical analysis

Data were analyzed using SPSS. Continuous variables were summarized as mean ± SD or median (range), and categorical variables as frequencies and percentages. Medical history and concomitant medications were summarized for the safety population. The primary efficacy outcome, change in mean sperm count from baseline to end of therapy, was analyzed using paired and unpaired Student’s t-tests. Secondary outcomes, including changes in sperm parameters, semen characteristics, and testosterone levels, were analyzed similarly. Nonparametric tests (Wilcoxon signed-rank, Mann-Whitney U, or chi-square test) were used for IIEF domain scores (erectile function, intercourse satisfaction, overall satisfaction). All tests were two-sided, with p < 0.05 considered statistically significant.

Methodology of PDE5 inhibition assay

The in vitro PDE5 inhibitory activity of the test formulation (SHL 1066) was evaluated using recombinant human PDE5 enzyme (Sigma-Aldrich, Cat# E9034). The enzyme was diluted 1:100 in assay buffer consisting of 50 mM Tris-HCl (pH 8.0) and 100 mM MgCl₂. A fresh enzyme solution was prepared on the day of the assay.

A 10 mg/mL stock solution of the musk tablet was prepared in distilled water. Different volumes (ranging from 2.5 μL to 25 μL) of this solution were tested to determine dose-dependent inhibition. The assay procedure was adapted from Lin et al. with minor modifications (24).

The reaction mixture (total volume: 500 μL) comprised 50 mM Tris buffer (pH 8.0), 100 mM MgCl₂, 7.5 μL of the enzyme solution, and varying amounts of the test solution. The mixture was pre-incubated at 37°C for one hour. Following this, 3 μM cyclic guanosine monophosphate (cGMP), freshly prepared in water, was added to initiate the reaction.

The final optimized assay conditions were as follows: 0.05 μg/mL PDE5A1, 1.14 μg/mL cGMP, and a 180-minute incubation at 37°C. After the reaction, the mixture was heated at 100°C for five minutes to terminate enzyme activity. Samples were cooled to room temperature prior to chromatographic analysis.

Chromatographic analysis

**Figure 2:**
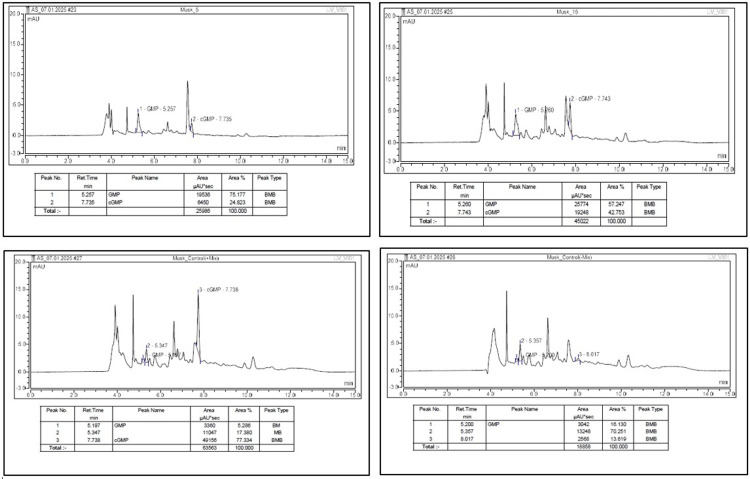
Chromatographic analysis of PDE5 inhibition assay PDE5 inhibitors: phosphodiesterase type 5 inhibitors

Following the enzymatic reaction, all samples were heat-inactivated at 100 °C for five minutes and cooled before being subjected to high-performance liquid chromatography (HPLC) analysis for quantification of residual cGMP. The HPLC system used was Waters Alliance e2695, equipped with a photodiode array detector and operated via Chromeleon 7 software. Separation was achieved using an Inertsil ODS C18 column (150 × 4.6 mm, 3.5 µm) maintained at 40°C, with the sampler temperature set at 15°C. The injection volume was 50 µL, and the detection wavelength was 254 nm. The mobile phases were as follows: Mobile Phase A-0.05 M potassium dihydrogen phosphate (prepared by dissolving 6.8 g of KH₂PO₄ in 1000 mL distilled water), and Mobile Phase B-methanol. Water or buffer was used as the diluent. The method utilized a gradient elution profile with a flow rate ranging from 0.6 to 1.5 mL/min. Clear separation of analytes was achieved with GMP and cGMP eluting at retention times of 5.3 minutes and 7.8 minutes, respectively. The total run time was 15 minutes (Figure 2). 

## Results

Demographic details

In the test group, the mean age was 37.80 + 04.94 years, whereas for the placebo group it was 36.80 + 04.77 years. The age range for participants was 29 to 45 years and 24 to 46 years, respectively.

Changes in the mean sperm cells between the groups

In the test subjects, the mean sperm cells at baseline visit were 08.10 ± 3.28, which significantly increased to 13.97 ± 2.20 (72.5% increase) at the end of the study. In the placebo subjects, the mean sperm cells at baseline visit were 07.67 ± 3.18, which significantly increased to 08.33 ± 1.54 (8.6% increase) at the end of the study. When compared between the groups, the test group performed significantly better than the placebo group at the end of the study. The details are presented in Table 1.

**Table 1:** Changes in the mean sperm cells, total motile sperm, and mean semen volume between the groups *indicates significant value within group; NS: not significant.​​​​​ The data are represented as mean ± S.D. and analysed using the Student T-test. Significant at p < 0.05

Duration (days)	Test	Placebo	P value	t value
Mean sperm cells (million/ml)				
Baseline	08.10 ± 3.28	07.67 ± 3.18	0.696 (NS)	0.52
Day 90	13.97 ± 2.20	08.33 ± 3.00	-	
Mean diff (baseline-90) (p-value)	*05.87 ± 3.09 (0.001)	*0.66 ± 1.54 (0.025)	0.001	8.27
Mean total motile sperm (%)				
Baseline	28.74 ± 10.11	27.66 ± 11.41	0.771 (NS)	0.39
Day 90	32.89 ± 09.01	29.41 ± 09.53	-	
Mean diff (baseline-90) (p-value)	*04.15 ± 6.36 (0.002)	01.76 ± 06.68 (0.166) NS	0.401 (NS)	1.42
Mean semen volume (ml)				
Baseline	01.67 ± 00.30	01.58 ± 00.35	0.478 (NS)	1.07
Day 90	01.78 ± 00.25	01.78 ± 00.47	-	
Mean diff (baseline-90) (p-value)	*00.12 ± 00.25 (0.013)	*00.20 ± 00.47 (0.026)	0.561 (NS)	-0.82

Changes in the mean total motile sperm (sperm motility) between the groups

In the test group, the mean total motile sperm count (sperm motility) at baseline was 28.74 ± 10.11, which showed a significant increase to 32.89 ± 9.01 by the end of the study, representing a 14.4% improvement. In contrast, the placebo group exhibited a nonsignificant increase from 27.66 ± 11.41 at baseline to 29.41 ± 9.53 at the end of the study, corresponding to a 6.3% increase. While the change in sperm motility was greater in the test group compared to the placebo group, the between-group difference was not statistically significant. Detailed data are provided in Table 1.

Changes in the mean semen volume between groups

In the test group, the mean semen volume at baseline was 1.67 ± 0.30 ml, which significantly increased to 1.78 ± 0.25 ml by the end of the study. Similarly, in the placebo group, the mean semen volume increased significantly from 1.58 ± 0.35 ml at baseline to 1.78 ± 0.47 ml at the end of the study. However, when compared between the groups, no significant difference was observed. The detailed findings are presented in Table 1.

Changes in the mean serum total testosterone between the groups

In test subjects, the mean serum total testosterone at baseline visit was 349.50 ± 120.46, which significantly increased to 430.80 ± 123.22 (23.3% increase) on day 90. In the placebo subjects, the mean serum total testosterone at baseline visit was 316.11 ± 194.46, which insignificantly increased to 328.51 ± 204.74 (3.9% increase) on day 90. If compared between the groups, the test group performed significantly better than the placebo group at the end of the study. The details are presented in Table 2.

**Table 2:** Changes in the mean serum total testosterone levels between the groups *indicates significant value within group; NS: not significant​​​​ The data is represented as mean ± SD and analyzed by the Wilcoxon Signed Rank Test (within group), Mann-Whitney U Test (between groups). Significant at p < 0.05

Duration (days)	Mean serum total testosterone		p-value	U value
	Test	Placebo		
Baseline	349.50 ± 120.46	316.11 ± 194.46	0.512 (NS)	405
Day 90	430.80 ± 123.22	328.51 ± 204.74	-	-
Mean diff (baseline-90 days) (p-value)	*81.30 ± 68.71 (0.001)	12.40 ± 67.33 (0.246) NS	0.001	227

Changes in mean erectile function between the groups

In the test subjects, the mean erectile function score on IIEF at baseline visit was 13.97 ± 1.69, which significantly increased to 16.03 ± 4.37 (14.8% increase), 16.83 ± 3.63 (20.5% increase), and 20.33 ± 3.02 (45.6% increase) on day 30, day 60, and day 90, respectively. In placebo subjects, the mean erectile function score on IIEF at baseline visit was 14.03 ± 2.53, which significantly increased to 16.40 ± 2.54 (16.9% increase), 14.77 ± 1.57 (5.2% increase), and 16.40 ± 2.25 (16.9% increase) on day 30, day 60, and day 90, respectively. If compared between the groups, the test performed significantly better than the placebo on days 60 and 90. The details are presented in Table 3.

**Table 3:** Changes in mean erectile function, intercourse satisfaction, and overall satisfaction between the groups *indicates significant value within group; NS: not significant​​​​​​ The data is represented as mean ± SD and analyzed by the Wilcoxon Signed Rank Test (within group), Mann-Whitney U Test (between groups). Significant at p < 0.05

Duration (days)	Test	Placebo	p-value (between)	U value
Mean erectile function				
Baseline	13.97 ± 1.69	14.03 ± 2.53	0.390 (NS)	415
Day 30	*16.03 ± 4.37	*16.40 ± 2.54	0.984 (NS)	446
Day 60	*16.83 ± 3.63	*14.77 ± 1.57	0.003	250
Day 90	*20.33 ± 3.02	*16.40 ± 2.25	0.001	190
Mean intercourse satisfaction				
Baseline	6.50 ± 0.90	6.47 ± 1.04	0.610 (NS)	398
Day 30	*8.63 ± 1.56	7.00 ± 1.76	0.000	160
Day 60	*7.83 ± 2.61	6.37 ± 0.93	0.059 (NS)	320
Mean overall satisfaction				
Baseline	4.20 ± 0.61	4.27 ± 0.69	0.406 (NS)	405
Day 30	*5.57 ± 1.19	4.83 ± 1.12	0.004	270
Day 60	*5.23 ± 1.87	4.33 ± 0.71	0.024	290
Day 90	*6.47 ± 1.46	*4.97 ± 1.22	0.001	190

Changes in the mean intercourse satisfaction between the groups

In test subjects, the mean intercourse satisfaction score on IIEF at baseline visit was 6.50 ± 0.90, which significantly increased to 8.63 ± 1.56 (32.8% increase), 7.83 ± 2.61 (20.5% increase), and 9.93 ± 2.08 (52.8% increase) on day 30, day 60, and day 90, respectively. In placebo subjects, the mean intercourse satisfaction score on IIEF at baseline visit was 66.47 ± 1.04, which nonsignificantly increased to 7.00 ± 1.76 (8.2% increase) on day 30. The mean intercourse satisfaction score on IIEF nonsignificantly reduced from baseline visit to 6.37 ± 0.93 (1.5% reduction) on day 60. The mean intercourse satisfaction score on IIEF significantly increased from baseline visit to 7.73 ± 1.78 (19.6% increase) on day 90. If compared between the groups, the change was significantly more among the test group than the placebo group on day 30 and day 90. The details are presented in Table 3.

Changes in the mean overall satisfaction between the groups

In the test subjects, the mean overall satisfaction score on IIEF at baseline visit was 4.20 ± 0.61, which significantly increased to 5.57 ± 1.19 (32.6% increase), 5.23 ± 1.87 (24.5% increase), and 6.47 ± 1.46 (54.0% increase) on day 30, day 60, and day 90, respectively. In the placebo subjects, the mean overall satisfaction score on IIEF at baseline visit was 4.27 ± 0.69, which nonsignificantly increased to 4.83 ± 1.12 (13.1% increase), 4.33 ± 0.71 (1.4% increase) on day 30 and day 60, respectively. The mean overall satisfaction score on IIEF significantly increased from baseline visit to 4.97 ± 1.22 (16.4% increase) on day 90. If compared between the groups, the change was significantly better among the test group than the placebo group on day 90. The details are presented in Table 3.

Safety assessment

Assessment of Compliance and Tolerability

Drug compliance was high in both the test and placebo groups, with no significant difference, while tolerability, as assessed by physicians and subjects, was significantly higher in the test group compared to the placebo group.

Profile of Adverse Events

In the test group, six (20%), subjects reported a total of six adverse events during the study period. These adverse events included a burning throat, burning micturition, and mild fever. All these adverse events were mild to moderate in severity. These adverse events were resolved completely after rescue medication was given. Study treatment was not stopped during these adverse events. All these adverse events were not related to the study drug. In the placebo group, three (10%) subjects reported a total of three adverse events during the study period. These adverse events included dyspepsia, dry cough, and loose motion. All these adverse events were mild to moderate in severity and resolved completely after rescue medication was given.

Physiological Profile Assessment

At baseline, both groups exhibited normal hemogram, lipid, liver, renal, and urine analysis profiles, as well as pulse rate, body temperature, and respiratory rate. Post-treatment, no significant changes were observed in these laboratory and vital parameters in either the Test or Placebo group.

In vitro PDE5 inhibitory activity

**Figure 3:**
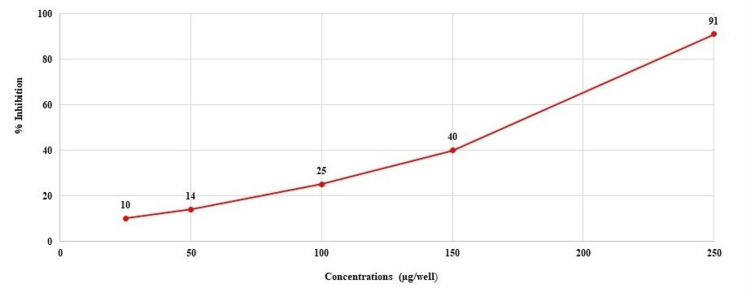
Dose-dependent inhibition at various concentrations

The investigational formulation demonstrated a concentration-dependent inhibition of PDE5 enzyme activity. As shown in Figure 3, increasing concentrations of the formulation resulted in progressively higher levels of enzyme inhibition, with a maximum inhibition of 91% observed at 250 µg/well.

## Discussion

The findings of this randomized, placebo-controlled study demonstrate that SHL 1066 significantly improves seminal parameters and reproductive hormone profiles in men with low sperm count. After 90 days of supplementation, subjects receiving SHL 1066 showed a marked 72.5% increase in sperm count and a 14.4% improvement in sperm motility, compared to only 8.6% and 6.3% increases, respectively, in the placebo group. These results suggest that SHL 1066 may be beneficial in addressing both oligozoospermia and asthenozoospermia. However, the between-group difference did not reach statistical significance. Also greater proportion of participants in the SHL 1066 group reached this threshold compared with placebo, suggesting a meaningful clinical benefit beyond statistical improvement. SHL 1066 also resulted in a 23.3% increase in serum total testosterone, reflecting improved testicular hormonal function.

In addition to improving fertility parameters, SHL 1066 significantly enhanced sexual health outcomes. Erectile function scores increased by 45.5%, with intercourse satisfaction and overall satisfaction improving by 52.8% and 54%, respectively, markedly higher than improvements seen with placebo. These enhancements in sexual function further support the clinical relevance of SHL 1066 in men experiencing subfertility accompanied by sexual dysfunction. Overall, the results indicate that SHL 1066 is a safe and effective therapeutic option for improving male fertility and sexual health. However, the linkage between in vitro PDE5 inhibition and in vivo sexual performance outcomes remains inferential, based on alignment with the known physiology of the nitric oxide-cGMP pathway. Further studies with larger sample sizes and extended follow-up periods are warranted to confirm these findings and explore long-term benefits.

A previous study on *Asparagus adscendens Roxb.* root extract demonstrated its efficacy in enhancing reproductive and sexual functions, with significant improvements observed in body and testicular weights, daily sperm production, epididymal sperm count, and sexual behavior parameters such as mounting frequency and ejaculation latency. These outcomes suggest a strong pharmacological basis for its traditional use in managing sexual debility and seminal weakness. The findings are consistent with the observed clinical benefits of SHL 1066 in the current study, further supporting the role of *Asparagus adscendens* as a key herbal component with potential in improving male reproductive health [24].

A previous systematic review critically evaluated evidence from randomized clinical trials to assess the efficacy of *Withania somnifera* (ashwagandha) in improving male sexual health outcomes. After an extensive search across major scientific databases, five studies met the inclusion criteria and were assessed to have a low risk of bias. Of these, four studies demonstrated significant improvements in male sexual health parameters, indicating the potential of *Withania* *somnifera *as a beneficial supplement. These findings align with our study, in which SHL 1066, containing *Withania somnifera* as a key ingredient, demonstrated a positive impact on sexual function and satisfaction scores in men with low sperm counts [25].

A previous randomized, double-blinded, placebo-controlled study assessed the effect of *Withania somnifera* root extract in adult males with low sexual desire. After eight weeks of supplementation (300 mg twice daily), participants showed a significant improvement in Derogatis Interview for Sexual Functioning (Male version, DISF-M) scores and serum testosterone levels. These results highlight ashwagandha’s potential in enhancing male sexual function and hormonal balance. Similar outcomes observed in our trial further support the growing evidence for herbal supplementation in male sexual health management [26].

A previous randomized, double-blinded, placebo-controlled clinical trial involving 180 men aged 18 to 65 years demonstrated the effectiveness of* Tribulus terrestris *(marketed as Tribestan®) in improving sexual function in males with mild to moderate ED, with or without hypoactive sexual desire disorder. Participants received 750 mg daily of *Tribulus terrestris *for 12 weeks, resulting in a statistically significant improvement in International Index of erectile function scores compared to placebo, with notable enhancements in intercourse satisfaction, orgasmic function, sexual desire, and overall satisfaction. The therapy was well accepted, with no serious adverse effects linked to the drug. These findings align with the outcomes of our study, where improvements in sexual desire, satisfaction, and overall sexual well-being were also observed, supporting the potential role of *Tribulus terrestris *in enhancing male sexual health [27].

A previous study on *Asparagus racemosus* willd., an established Vajikaran rasayana herb in Ayurveda, demonstrated its efficacy in reversing diabetes-induced sexual dysfunction in rats. The polysaccharide-rich root extract, high in 2→1 type fructooligosaccharides, significantly improved sexual behavior parameters such as mount, ejaculation, and intromission frequencies by counteracting oxidative stress. These findings support its traditional use as an aphrodisiac. This is in line with our study, where *Asparagus racemosus*, included in the formulation SHL 1066, showed a positive contribution to male sexual and reproductive health outcomes [28].

A previous study investigated the spermatotoxic effects of ochratoxin in male mice and its mitigation by *Emblica officinalis (amla)* aqueous extract. Administration of ochratoxin significantly impaired sperm parameters such as count, motility, viability, and fertility rate, whereas co-treatment with *Emblica officinalis* led to a marked improvement in all these parameters. The extract alone did not affect reproductive outcomes, confirming its safety and potential as a protective agent. These findings underscore the antioxidant and reproductive protective properties of *Emblica officinalis,* which are consistent with our clinical study results where *Emblica officinalis *was a constituent of the tested formulation that showed notable improvements in male reproductive parameters, including sperm count and motility [29].

The L-arginine-nitric oxide-cGMP pathway is crucial for penile erection and smooth muscle relaxation, with PDE5 breaking down cGMP to control it. Our in vitro study aligns with this mechanism, demonstrating that the polyherbal formulation effectively inhibits human PDE5, potentially amplifying the cGMP pathway [30].

This research has several strengths that add to the credibility and relevance of its findings. It was thoughtfully designed as a randomized, placebo-controlled study conducted across multiple centers, enhancing the overall reliability and generalizability of the results. SHL 1066 demonstrated clear and statistically significant improvements in sperm count, motility, testosterone levels, and sexual performance in men with low sperm count. The formulation includes well-established herbal ingredients such as *Withania somnifera, Asparagus adscendens, Asparagus racemosus, Tribulus terrestris, and Emblica officinalis*, each of which has a history of traditional use and scientific support for improving male reproductive health. The consistency of this study’s outcomes with previous research further validates the efficacy of SHL 1066. However, some limitations should be noted. The relatively small sample size and limited study duration of 90 days may not fully capture the long-term effects of the intervention. Additionally, the study did not assess semen morphology or explore the underlying biological mechanisms in depth. Future research involving larger populations, extended treatment periods, and mechanistic evaluations with hormonal intermediates, oxidative-stress biomarkers, or other pathways may help to strengthen and expand upon these findings. 

## Conclusions

SHL 1066 demonstrated significantly greater efficacy in improving key semen parameters, compared to placebo, including the total number of sperm cells per milliliter of seminal fluid and sperm motility. Furthermore, the formulation led to notable improvements in sexual health outcomes, with significant enhancements observed in erectile function, intercourse satisfaction, and overall satisfaction scores. Across the 90-day study period, the formulation was generally well-tolerated. Though participants experienced mild, nondrug-related adverse events, all events resolved without treatment discontinuation. The findings of this study suggest that SHL 1066 is both a safe and effective therapeutic option for men with low sperm count. Its dual action on improving semen quality and sexual function supports its potential utility in the comprehensive management of male infertility.
